# Doing Pre-operative Investigations in Emergency Department; a Clinical Audit

**Published:** 2017-01-09

**Authors:** Muhammad Salman Rafiq, Maria Rafiq, Muhammad Imran Rafiq, Seema Gul Salman, Sania Hafeez

**Affiliations:** 1 Department of Surgery, Khyber Teaching Hospital, Peshawar, Pakistan.; 2 Department of Obstetrics and Gynecology, Khyber Teaching Hospital, Peshawar, Pakistan.; 3 Department of Cardiology, Khyber Teaching Hospital, Peshawar, Pakistan.; 4 Department of Obstetrics and Gynecology, Mardan Medical Complex, Mardan, Pakistan.; 5 Department of Obstetrics and Gynecology, Hayatabad Medical Complex, Peshawar, Pakistan.

**Keywords:** Clinical audit, management audit, emergency treatment, emergencies

## Abstract

**Introduction:**

Pre-operative investigations for emergency surgical patients differ between centers. Following established guidelines can reduce unnecessary investigation, cost of treatment and hospital stay. The present audit was carried out to evaluate the condition of doing pre-operative investigations for three common surgical emergencies compared to National Institute for Health and Care Excellence (NICE) guidelines and local criteria.

**Methods:**

A retrospective clinical audit of acute-appendicitis, abscess and hernia patients admitted to the emergency department was carried out over a one-year period from July 2014 to July 2015. Data of laboratory investigations, their indication, cost and duration of hospital stay was collected and compared with NICE-guidelines.

**Results:**

A total of 201 patients were admitted to the emergency department during the audit period. These included 77(38.3%) cases of acute-appendicitis, 112 (55.7%) cases of abscesses, and 12 (6%) cases of hernia. Investigations not indicated by NICE-guidelines included 42 (20.9%) full blood counts, 29 (14.4%) random blood sugars, 26 (12.9%) urea tests, 4 (2%) chest x-rays, 13 (6.5%) electrocardiographs, and 58 (28.9%) urine analyses. These cost 25,675 Rupees (245.46 Dollars) in unnecessary investigation costs and 65.7 days of additional hospital stay.

**Conclusions:**

Unnecessary investigations for emergency surgical patients can be reduced by following NICE-guidelines. This will reduce workload on emergency services, treatment costs and the length of hospital stay.

## Introduction

Surgical management is dependent upon laboratory and radiological investigations, and treatment guidelines and scoring systems usually rely on their findings (1). These investigations are associated with some adverse outcome (2). All radiological evaluations expose subjects to variable doses of radiation and may require contrast, which is associated with its own adverse effects. On the other hand, hematological investigations are associated with the risk of infection transmission, needle stick, hemorrhage, and etc. (3). Whereas laboratory investigations are relatively cheaper and are usually prescribed by junior medical personnel, radiological ones are costly and are usually prescribed by consultants or senior medical personnel. There are no fixed guidelines of prescription for these investigations in most centers. These differences are responsible for investigations being performed in large numbers, more frequently, and in some cases, unnecessarily (4). In an emergency department, investigation services are usually being utilized at any given instance. These slots are simultaneously used by various departments in addition to emergency surgical services. A careless attitude towards laboratory investigations will result in unnecessary investigations, increase in treatment costs, straining of laboratory services, increase in hospital stay and patient mismanagement. Such practices are more prevalent in the absence of guidelines (5). Presence and implementation of guidelines outlining investigations for any given condition reduces such unnecessary testing. This reduces the cost of treatment as shown by previous studies (5, 6). International and local guidelines specifically created for pre-operative emergency surgical management are lacking. The National Institute for Health and Care Excellence (NICE) guidelines for pre-operative management of surgical patients were created for elective cases (7). These can be applied to emergency surgeries because they are based on a simple grading of surgical procedures. Based on the above mentioned, the present audit was carried out to evaluate the condition of doing pre-operative investigations for three common surgical emergencies compared to National Institute for Health and Care Excellence (NICE) guidelines and local criteria.

## Methods

***Study design***

*A *retrospective clinical audit of 201 randomly selected patients of three common surgical emergencies, admitted to the emergency department of Khyber teaching Hospital, was carried out over a one-year period from July 2014 to July 2015. These included patients of acute appendicitis, acute hernia and abscesses requiring emergency surgery. Approval of the hospital’s ethical and research committees was obtained.

***Participants***

All patients over the age of 18 years of both male and female gender with diagnosis of acute appendicitis, abscess or hernia presenting to emergency department and requiring emergency surgery were included.

***Data gathering***

Data was collected, using a printed preform, from the treatment charts and emergency outpatient sheets. Data of laboratory and radiological investigations carried out for the three mentioned surgical emergencies was collected for each patient. Indicated investigations by NICE guidelines and local criteria were determined and inappropriate investigations were defined as investigations that were not indicated or performed more than once without reason. If investigations were inappropriately carried out, the resultant cost and increase in duration of hospital stay were calculated. 

Since local criteria and NICE guidelines differed in all instances for indication of each pre-operative emergent investigation, data regarding indication of doing investigation in both instances was collected. Local criteria include a total of 12 investigations compared to 6 investigations of the NICE guidelines. An additional NICE investigation i.e. hemostasis was not made part of pre-operative testing as it was not indicated in any of the cases. These are detailed below in audit standards and local criteria sections.

*Audit standards*

In 2003, NICE published guidelines for the routine pre-operative investigations of elective surgery (7). These guidelines were used as the standards for comparison in the present study (panel 1 to 3). These guidelines declare the indications of seven commonly performed investigations include: chest x-ray; electrocardiography; complete blood count; hemostasis profile [Bleeding time, clotting time, platelets count, prothrombin time (PT), partial thromboplastin time (PTT), international normalized ratio (INR)]; renal function tests (Urea, creatinine); random blood sugar; and urine analysis. The guidelines include hemostasis as a required investigation but it was not included in this audit where pre-operatively hemostasis assessment is rarely indicated for the studied surgical emergencies. 

For abscesses, which require surgical incision and drainage, categorized as grade 1 or minor surgery, investigations should include as shown in panel 1. 

For cases of right lower quadrant pain clinically diagnosed as acute appendicitis or cases presenting as acute obstructed or strangulated hernia, requiring emergency surgery, both in the category of grade 2 or intermediate surgery, investigations should include as shown in panel 2. 

For cases of acute obstructed or strangulated hernia, requiring emergency surgery, considered as grade 3 or major surgery, investigations should include as shown in panel 3.

For acute hernia, major surgery was defined as cases clinically determined pre-operatively with a strong possibility of bowel gangrene or requiring resection and anastomosis. In each instance, a pre-operative test should be sampled and the result obtained only once, unless it is inadequately sampled or reported or there is doubt about the result requiring a repeat sampling and testing. 

*Local criteria (hospital guideline)*

Based on our local criteria pre-operative management of the three mentioned surgical emergencies included 12 investigations. These investigations and their costs were as follows: complete blood count (CBC), 45 Rupees (43 Cents); random blood sugar (RBS), 45 Rupees (43 Cents); urea, 45 Rupees (43 Cents); serum electrolytes, 100 Rupees (96 Cents); liver function tests (LFTs), 120 Rupees (1.15 Dollar); serum amylase, 45 Rupees (43 Cents); C-reactive protein (CRP), 200 rupees (1.91 Dollar); ultrasonography (USG), 220 Rupees (2.1 Dollars); chest x-ray with non-digital device (CXR), 50 Rupees (48 Cents); electrocardiogram (ECG), 50 Rupees (48 Cents); viral markers (non-ELISA including Hepatitis B, C and HIV), 100 Rupees (96 Cents); and urine analysis (U/A), 45 Rupees (43 Cents).

For abscesses, which require surgical incision and drainage, investigations should include:

- Complete blood count, viral markers, and if indicated chest x-ray, ECG, ultrasonography of abscess cavity for patients younger than 40 years
- Complete blood count, viral markers, chest x-ray, ECG, and if indicated RBS, renal function, electrolytes and ultrasonography of abscess cavity for patients ≥ 40 years

For cases of right lower quadrant pain clinically diagnosed as acute appendicitis requiring emergency surgery, investigations should include:

- Complete blood count, viral markers, ultrasonography of abdomen and pelvis for patients younger than 40 years
- Complete blood count, viral markers, ultrasonography of abdomen and pelvis, chest x-ray and ECG for patients ≥ 40 years

For cases presenting as acute obstructed or strangulated hernia, requiring emergency surgery, investigations should include:

- Complete blood count, viral markers, ultrasonography of abdomen and pelvis, urea, serum electrolytes, and if indicated Chest and/or Abdominal x-ray, ECG, and serum amylase for patients younger than 40 years
- Complete blood count, viral markers, ultrasonography of abdomen and pelvis, urea, serum electrolytes, chest x-ray, ECG, and if indicated serum amylase for patients ≥ 40 years

Serum Amylase is prescribed to rule out gastro intestinal pathology such as pancreatitis, hollow viscus perforation and other causes.

***Statistical analysis***

All data were recorded in the statistical software SPSS version 20. Comparison was made with NICE guidelines and local criteria for laboratory and radiological investigations for acute appendicitis, abscesses and hernia requiring emergency surgery using chi square or Fisher’s exact tests. Data were expressed as frequency and percentage or mean ± standard deviation.

## Results

A total of 201 patients were analyzed. The mean age of the patients was 48.26 ± 18.72 years. There were 77 (38.3%) cases of acute appendicitis, 112 (55.7%) cases of abscesses and 12 (6%) cases of acute hernia. Condition of doing preoperative investigations based on local criteria and National Institute for Health and Care Excellence (NICE) guidelines is shown in table 1. RBS was the most in-appropriately performed investigation with 48 (23.9%) cases where it was performed twice pre-operatively, followed closely by CBC in 38 (18.9%) cases. Viral markers were checked for all cases whereas CRP was not performed in any case. The greatest difference between local and NICE guideline was for CBC; 173 (86.1%) cases as per local criteria compared to only 42 (20.9%) cases per NICE guidelines.

***Patient’s Cost***

CBC was responsible for the largest cost of 8,415 Rupees (80.45 Dollars) for inappropriate testing. Inappropriate testing leading to additional costs for RBS, Urea, CXR, ECG and urine analysis was calculated at 4950 Rupees (47.32 Dollars), 3195 Rupees (30.55 Dollars), 2250 Rupees (21.51 Dollars), 4300 Rupees (41.11 Dollars) and 2565 Rupees (24.52 Dollars., respectively. The total cost for all of these was 25,675 Rupees (245.46 Dollars).

***Duration of hospital stay***

CBC was responsible for the largest contribution to unnecessary hospital stay at 41390 minutes, which equals to a total of 28.7 days. The smallest contribution to unnecessary hospital stay was due to CXR at 2565 minutes or 1.8 days. The total number of unnecessary hospital stay was calculated to be 94550 minutes (65.7 days). 

## Discussion

Clinical practice guidelines are end results of large multi-center studies and multi-disciplinary approaches (8). The NICE guidelines, related to clinical practice, were created after multiple studies including clinical audits (7). This resulted in the proposal of the 2003 NICE guideline for the pre-operative assessment of surgical patients. Originally, these guidelines were created for elective surgery cases but they can be applied to emergency surgeries as well. This is because the NICE guidelines are classified on a simple grading of surgeries as Grade 1 to 4 (7).

Health system in Pakistan is not organized on central uniform guidelines. Tertiary care hospitals usually follow local criteria, foreign clinical guidelines or a combination of both. However, no strict guidelines are followed for doing preoperative investigation for surgical emergencies. Local practices for emergency surgery, such as acute appendicitis, abscesses requiring surgical treatment and obstructed or strangulated hernias, are not outlined (9). 

**Panel 1:** Required pre-operative investigations for grade 1 or minor surgeries

**Investigations**	**Age (Years)**			
	**18 to < 40**	**40 to < 60**	**60 to < 80**	**80 <**
**Chest x-ray**	No	No	No	No
**Electrocardiography**	No	Consider	Consider	Yes
**Complete blood count**	No	No	Consider	Consider
**Hemostasis profile**	No	No	No	No
**Renal Function tests**	No	No	Consider	Consider
**Random blood sugar**	No	No	No	No
**Urine analysis**	Consider	Consider	Consider	Consider

**Panel 2:** Required pre-operative investigations for grade 2 or intermediate surgeries

**Investigations**	**Age (Years)**			
	**18 to < 40**	**40 to < 60**	**60 to < 80**	**80 <**
**Chest x-ray**	No	No	No	No
**Electrocardiography**	No	Consider	Consider	Yes
**Complete blood count**	No	Consider	Yes	Yes
**Hemostasis profile**	No	No	No	No
**Renal Function tests**	No	No	Consider	Consider
**Random blood sugar**	No	Consider	Consider	Consider
**Urine analysis**	Consider	Consider	Consider	Consider

**Panel 3:** Required pre-operative investigations for grade 3 or major surgeries

**Investigations**	**Age (Years)**			
	**18 to < 40**	**40 to < 60**	**60 to < 80**	**80 and >**
**Chest x-ray**	No	No	Consider	Consider
**Electrocardiography**	No	Consider	Yes	Yes
**Complete blood count**	Yes	Yes	Yes	Yes
**Hemostasis profile**	No	No	No	No
**Renal Function tests**	Consider	Consider	Yes	Yes
**Random blood sugar**	Consider	Consider	Consider	Consider
**Urine analysis**	Consider	Consider	Consider	Consider

**Table 1:** Condition of doing preoperative investigations based on local criteria and National Institute for Health and Care Excellence (NICE) guidelines

**Investigation**	**Local criteria**			**NICE guidelines**		
	Indicated	Performed	#Inappropriate	Indicated	Inappropriate cost Rupees(Dollar)	Additional stay (minutes)
**CBC**	173(86.1%)	173(86.1%)	38(18.9%)	42(20.9%)	8,415 (80.45)	41390
**RBS**	82(40.8%)	82(40.8%)	48(23.9%)	29(14.4%)	4,950 (47.32)	20140
**Urea**	82(40.8%)	82(40.8%)	15(7.5%)	26(12.9%)	3,195 (30.55)	17490
**Electrolytes**	21(10.4%)	21(10.4%)	12(6%)			
**LFTs**	1(0.5%)	1(0.5%)	0(0%)			
**Amylase**	9(4.5%)	9(4.5%)	0(0%)			
**CRP**	0(0%)	0(0%)	0(0%)			
**Sonography**	123(61.2%)	123(61.2%)	24(11.9%)			
**CXR**	38(18.9%)	38(18.9%)	2(1%)	4(2%)	2,250 (21.51)	2290
**ECG**	73(36.3%)	73(36.3%)	17(8.5%)	13(6.5%)	4,300 (41.11)	4490
**Viral profile**	201(100%)	201(100%)	32(15.9%)			
**Urine analysis**	91(45.3%)	91(45.3%)	11(5.5%)	58(28.9%)	2,565 (24.52)	8750
**Total**					25,675(245.46)	94550

CBC: complete blood count; RBS: random blood sugar; CRP: C reactive protein; CXR: chest x ray; ECG: electrocardiography.

* represents investigations which are not part of NICE guidelines.

# represents cases where investigations were ordered multiple times when indicated only once.

Differences were expected between local criteria and NICE guidelines. An important difference was the fewer number of investigations by NICE standards; seven compared to 12 for local criteria. NICE utilizes tests for hemostasis, but for the three surgical processes included in the audit, they are rarely required. NICE does not include some investigations in its guidelines, which are used in local criteria. These include electrolytes, LFT, amylase, CRP, viral markers and ultrasonography. These were included only as part of local criteria. The most important local criteria not included in NICE is viral markers. Local criteria requires that testing for Hepatitis B, C and HIV be carried out in every case (10).

The audit fell short in indication for various investigations. Based on local criteria, CBC was indicated in 173 (86.1%) cases, whereas it was only indicated in 42 (20.9%) cases based on NICE guidelines. This represents a difference of 131 cases or 65.2%. Difference between local and NICE guidelines for checking preoperative RBS, urea, CXR, ECG and urine R/E were 26.4%, 27.9%, 16.9%, 29.8% and 16.4%, respectively. 

In many cases, investigations were ordered multiple times when they were required only once for pre-operative assessment. RBS was the most commonly abused investigation, which was inappropriately performed in 48 (23.9%) cases. This was closely followed by CBC with 38 (18.9%) cases. Multiple investigation instances for urea, CXR, ECG and urine analysis were 15 (7.5%), 2 (1%), 17 (8.5%) and 11 (5.5%), respectively. Analysis of data showed that in all instances of multiple investigations, they were performed only twice.

***Cost***

Multiple instances and inappropriately indicated investigations based on NICE guidelines resulted in unnecessary expenditure to the hospital. The greatest cost of unnecessary expenditure was caused by CBC at 8,415 Rupees (80.45 Dollars). This was followed by RBS at 4,950 Rupees (47.32 Dollars). This resulted in a total loss of 25,675 Rupees (245.46 Dollars) of unnecessary investigations.

***Hospital stay***

Inappropriate testing was responsible for unnecessary and prolonged hospital stay. The largest contribution to unnecessary hospital stay was caused by CBC closely followed by RBS, urea, CXR, ECG, and urine analysis, respectively. The total time in terms of unnecessary hospital stay was calculated to be 94550 minutes (65.66 days).

There are a number of factors responsible for unnecessary investigations being ordered for emergency surgical patients. In emergency surgical management, almost all investigations are ordered by junior doctors and sometimes nurses who are not involved in clinical assessment or operative treatment. There are no evidence-based guidelines for investigation of emergency surgical patients. NICE guidelines were originally proposed for elective surgical cases. In many instances, investigations are repeated pre-operatively without a reason or by mistake. There are instances where these are repeated because first samples taken and reports provided are by external labs, which are not part of the hospital.

Investigations not related to the disease process and its management are responsible for a sizeable portion of the audit shortcomings. CBC might not be required in cases of simple incision and drainage where systemic signs are absent. Urea was ordered in young patients and cases of simple abscesses. Other investigations ordered for simple abscesses, which were not indicated by NICE guidelines, included ultrasonography, CXR, ECG and urine analysis. Cases where the presenting disease process was associated with abnormal incidental findings in other systems were responsible for some of these unnecessary investigations. This accounted for some of the cases where for example urea and electrolytes were ordered for abscess or LFTs, CXR and ECG for young patients of acute appendicitis, etc. This aspect requires a separate audit because it contributes to unnecessary investigations, cost and hospital stay.

Investigations, which are not part of NICE guidelines but are required by evidence-based recommendations for all admitted patients, require a separate audit. These include CRP and viral markers of all admitted patients for Hepatitis B, C and HIV. Serum amylase also falls in this category. 

Recommendations

 To overcome the shortcoming in the audit, the following recommendations are made:

- Posters about NICE guidelines for pre-operative management of surgical cases should be set up in the emergency department.
- Staff of the emergency department especially junior doctors and nurses should be educated about NICE guidelines and the findings of this audit through presentations.
- Investigations ordered for all cases should follow NICE guidelines.
- Investigations should not be repeated pre-operatively unless indicated.
- Investigations carried out in other setups before admission should be discussed with seniors to avoid multiple investigations.

## Conclusions

Based on the results of the present audit, investigations ordered without indication, and in some cases multiple times, were responsible for unnecessary treatment cost and prolonged hospital stay. Following NICE guidelines or establishing evidence-based local guidelines can reduce and prevent these mistakes and costs.
